# Cephalopod Tissue Regeneration: Consolidating Over a Century of Knowledge

**DOI:** 10.3389/fphys.2018.00593

**Published:** 2018-05-23

**Authors:** Pamela Imperadore, Graziano Fiorito

**Affiliations:** ^1^Association for Cephalopod Research - CephRes, Napoli, Italy; ^2^Department of Biology and Evolution of Marine Organisms, Stazione Zoologica Anton Dohrn, Napoli, Italy

**Keywords:** regeneration, wound healing, functional recovery, cephalopod, invertebrates

## Abstract

Regeneration, a process consisting in regrowth of damaged structures and their functional recovery, is widespread in several phyla of the animal kingdom from lower invertebrates to mammals. Among the regeneration-competent species, the actual ability to restore the full form and function of the injured tissue varies greatly, from species being able to undergo whole-body and internal organ regeneration, to instances in which this ability is limited to a few tissues. Among invertebrates, cephalopod mollusks retain the ability to regenerate several structures (i.e., muscles, nerves, or entire appendages). Here we provide an overview of more than one-hundred studies carried out over the last 160 years of research. Despite the great effort, many aspects of tissue regeneration in cephalopods, including the associated molecular and cellular machinery, remain largely unexplored. Our approach is largely descriptive and aims to provide a reference to prior work thus to facilitate future research efforts. We believe such research may lead to important discoveries and approaches that can be applied to other animal taxa including higher vertebrates, as well as other research fields such as regenerative medicine.

## Introduction

Johannes Japetus Steenstrup, a Danish zoologist (biography available in: Müller, 1976; Farley, 2001), was the first to report evidence for appendage regeneration in cephalopods. In his “*Hectocotyldannelsen hos Octopodslægterne Argonauta og Tremoctopus, oplyst ved Iagttagelse af lignende Dannelser hos Blæksprutterne i Almindelighed*” (Steenstrup, 1856), [which was translated into English one year later (1857)], Steenstrup provided a thorough description of how the hectocotylus is formed in species belonging to the *Argonauta* and *Tremoctopus* genera. The Author also provided a description of the ability of this arm to regenerate if lost during copulation.

These accounts appeared about 170 years after the first report of regenerative abilities in any animal (for review see Dinsmore, 1991).

By examining decades of scientific literature, we found accounts that provide evidence of regeneration occurring in a variety of cephalopod tissue types, including the appendages (arms and tentacles), as well as aspects of the peripheral and central nervous systems. It has been also observed in the fossil record (e.g., shell repair in Ammonoidea; Buckman, 1891; Keupp, 1976, 2000). Among many, Mathilde M. Lange was the first to both provide a detailed description of cephalopod tissue regeneration, and pioneered a new avenue of study through experimental lesioning of arms, tentacles, skin and nerves (Lange, 1920). Similar experimental studies of regeneration events occurring after lesioning of peripheral nervous structures, such as the pallial- and the stellar nerves (which control skin patterning and breathing movements), were performed later (e.g., Sereni, 1929b; Sereni and Young, 1932; Sanders and Young, 1974). Collectively, this work has contributed to our understanding of the connectivity between the central and peripheral nervous systems of cephalopods.

## Aims, organization and general outline of this review

Cephalopods offer a valuable system with which to study regeneration phenomena and their underlying physiological mechanisms. Such research may lead to important discoveries and approaches that can be applied to other animal groups (including higher vertebrates) as well as other research fields, such as regenerative medicine.

Our review is based on a survey of the scientific literature initiated through an index provided by the Zoological Record (ZR)^1^, including both library holdings (i.e., ZR-volume collection of the Stazione Zoologica Anton Dohrn) and ZR-modern e-databases, as well as a subsequent search for non-digitized references identified in these works.

An analysis of the indexed works in Zoological Record for the number of scientific publications from the last seventy years concerning “regeneration AND Mollusca” (excluding cephalopods) allowed us to identify about 50 published works out of a total of more than 13,000 references using “regeneration” as a topic.

**Figure 1:**
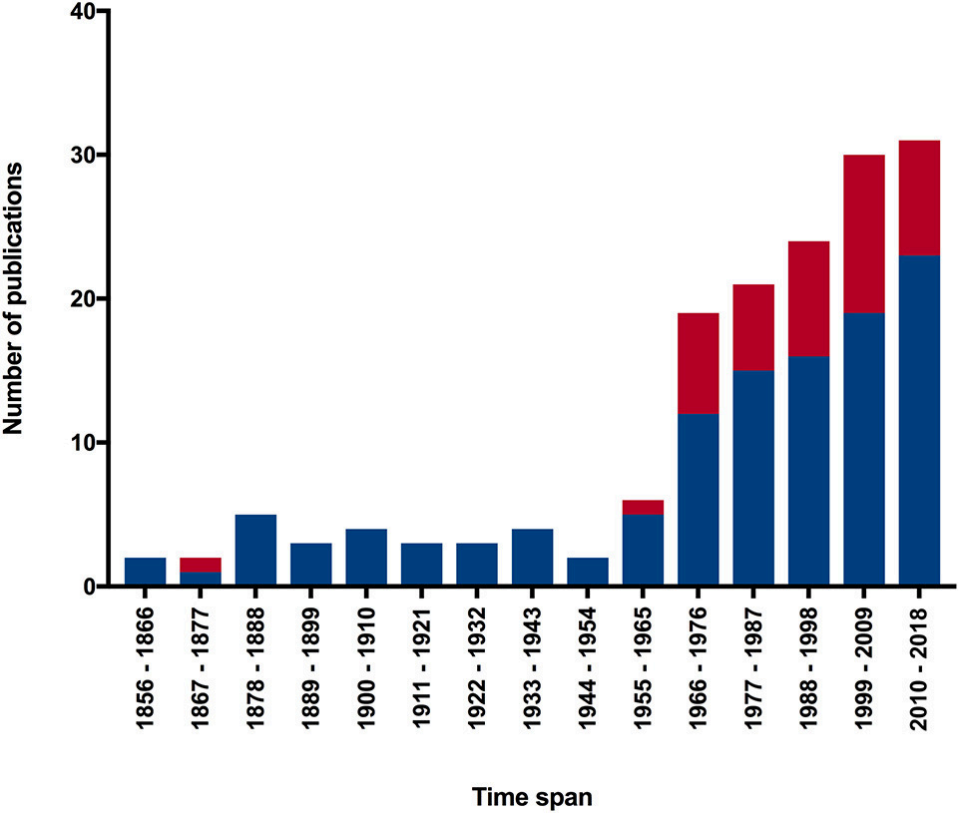
Trend of the number of publications regarding cephalopod regeneration from the first study published in 1856 to present. Number of indexed scientific works deduced from a query to Zoological Record (http://wokinfo.com/products_tools/specialized/zr/) concerning “regeneration AND cephalopod^*^.” The graph shows an enormous increase in published works in the last 50 years covering both living cephalopod tissue regeneration (blue) and fossil record shell repair (red). See text for further information.

By contrast, we identified around 120 works studying cephalopod regeneration (Figure 1) starting from Steenstrup's publication of 1857, as mentioned above. The figure shows a notable increase in the number of reports concerning the study of the regenerative phenomena in cephalopods over the last 50 years: this seemed especially pronounced over the last two decades (e.g., Rohrbach and Schmidtberg, 2006; Fossati et al., 2013, 2015; Tressler et al., 2014; Imperadore et al., 2017; Zullo et al., 2017).

Here, we summarize available knowledge of regeneration phenomena in cephalopod mollusks, providing an historical analysis of the studies carried out over the last 160 years on the regenerative abilities of the taxon.

Our approach is largely descriptive and aims to provide a convenient reference to prior work in order to facilitate future research efforts. The availability of new tools and approaches, as well as renewed interest in these complex invertebrates, may help in deciphering the molecular and cellular mechanisms involved in tissue regeneration, and could potentially inform our understanding of how the process can be dysregulated or inhibited in non-regenerating species.

**Figure 2:**
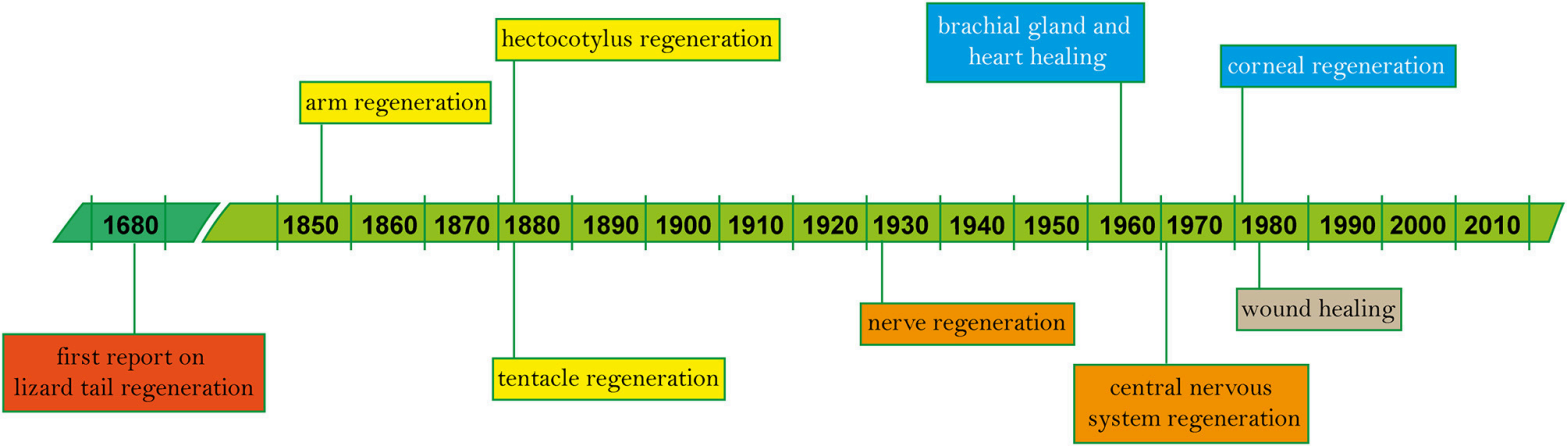
Timeline of regeneration in cephalopods. Since the first known records on vertebrate regeneration (i.e., lizard tail) by Thevenot in 1686 and Perrault account, 2 years later (Dinsmore, 1991), main findings on tissue regeneration in cephalopods based on published works are highlighted in chronological order (see Table 1 for the complete list).

The following pages offer a systematic overview of the findings described in a total of 119 works (Table 1) spanning the years 1856 to 2018, and a simplified outline of main discoveries (Figure 2). In addition to the tabularized overview of the regenerative process presented in Table 1, we also highlight first the events occurring in the early stages after damage (i.e., wound healing, both after skin injury and as first step of arm amputation), and second the ability of re-growing lost body parts, including regaining of function.

**Table 1:** A tabular overview of the studies of regeneration abilities of cephalopod molluscs.

**Main Topic Year of publication**	**General description**	**Species**	**Fossilrecord**	**Nautilus**	**Cuttlefish**	**Squid**	**Octopus**	**References**
**WOUND HEALING**								
1983	Skin healing	*Eledone cirrhosa* (Lamarck, 1798)					✓	Polglase et al., 1983
1988	Wound healing in the arm	*Sepia officinalis* (Linnaeus, 1758)			✓			Féral, 1988
2006	Skin healing	*Sepia officinalis* (Linnaeus, 1758)			✓			Harms et al., 2006
2016	Wound healing in the arm	*Octopus vulgaris* (Cuvier, 1797)					✓	Shaw et al., 2016
**ARM ABNORMALITIES**								
1893	Double hectocotylus	*Eledone cirrhosa* (Lamarck, 1798)					✓	Appellof, 1893
1898	Sub-numerary arms (seven) in octopus	*Enteroctopus megalocyathus* (Gould, 1852)					✓	Lönnberg, 1898
1900	Extra arm in octopus*;* arm branching in octopus	*Octopus vulgaris* (Cuvier, 1797); *Eledone cirrhosa* (Lamarck, 1798); *Eledone moschata* (Lamarck, 1798)					✓	Parona, 1900
1907	Arm branching in octopus	*Octopus cephea* (Gray, 1849) **taxon inquirendum**					✓	Smith, 1907
1913	Arm branching	*Octopus vulgaris* (Cuvier, 1797); *Eledone cirrhosa* (Lamarck, 1798); *Eledone moschata* (Lamarck, 1798)					✓	Hanko, 1913
1929	Double hectocotylus	*Octopus rugosus* (Bosc, 1792) **taxon inquirendum**					✓	Robson, 1929
1937	Arm branching	*Sepia esculenta* (Hoyle, 1885)			✓			Okada, 1937
1960	Arm branching	*Octopus briareus* Robson, 1929					✓	Kumpf, 1960
1965	Specimens of Japanese octopus with several branched arms	N/A					✓	Okada, 1965a
1965	Arm branching ”rules" in the Japanese octopus	N/A					✓	Okada, 1965b
1973	Double hectocotylus in octopus	*Octopus vulgaris* (Cuvier, 1797); *Octopus selene* (Voss, 1971)					✓	Palacio, 1973
1989	Sub-numerary arms (seven) in octopus	*Octopus* sp.						Gleadall, 1989
1991	Six-armed specimen (*Pteroctopus tetracirrhus*) 10-armed specimen (*Octopus briareus*)	*Pteroctopus tetracirrhus* (Delle Chiaje, 1830); *Octopus briareus* Robson, 1929					✓	Toll and Binger, 1991
2007	Double hectocotylus	*Octopus minor* (Sasaki, 1920)						Higashide et al., 2007
2013	Bilateral hectocotylization	*Enteroctopus dofleini* (Wülker, 1910)					✓	Brewer and Seitz, 2013
2014	Arm branching	*Octopus hubbsorum* (Berry, 1953)					✓	Alejo-Plata and Méndez, 2014
**ARM AUTOTOMY**								
1952	Arm autotomy*;* regeneration of lost structures is hypothesized	*Tremoctopus violaceus* (delle Chiaje, 1830)					✓	Portmann, 1952
1990	Automutilation syndrome in *Octopus dolfleini, O. bimaculoides*, and *O. maya*	*Enteroctopus dofleini* (Wülker, 1910); *Octopus bimaculoides* (Pickford & McConnaughey, 1949); *Octopus maya* (Voss & Solís, 1966)					✓	Reimschuessel and Stoskopf, 1990
1992	Arm autotomy	*Ameloctopus litoralis* Norman, 1992					✓	Norman, 1992
2001	Arm autotomy and regeneration; arm autotomy	*Abdopus capricornicus* (Norman & Finn, 2001) *Ameloctopus litoralis* Norman, 1992; *Octopus mutilans* (Taki, 1942)					✓	Norman and Finn, 2001
2012	Arm autotomy and regeneration	*Octopoteuthis deletron* Young, 1972				✓		Bush, 2012
**ARM REGENERATION**								
1856	Hectocotylus-formation in *Argonauta* and *Tremoctopus;* arm regeneration in *Octopus* sp.	N/A					✓	Steenstrup, 1856
1857	Hectocotylus-formation in *Argonauta* and *Tremoctopus;* arm regeneration in *Octopus* sp.	N/A					✓	Steenstrup, 1857
1881	Sucker, arm and tentacle regeneration in *Loligo pealei* and *Ommastrephes illecebrosus*	*Doryteuthis (Amerigo) pealeii* (Lesueur, 1821); *Illex illecebrosus* (LeSueur, 1821)				✓		Verrill, 1881
1881	Arm regeneration Shell aberration	*Octopus vulgaris* (Cuvier, 1797) *Sepia officinalis* (Linnaeus, 1758)			✓			Richiardi, 1881
1882	Arm regeneration in *Architeuthis harveyi*	*Architeuthis dux* Steenstrup, 1857				✓		Verrill, 1882
1901	Arm regeneration in *Octopus Defilippii*	*Macrotritopus defilippi* (Vérany, 1851)					✓	Riggenbach, 1901
1909	Arm autotomy and regeneration in *Octopus Defilippii*	*Macrotritopus defilippi* (Vérany, 1851)					✓	Lo Bianco, 1909
1916	Arm regeneration in *Polypus rugosus* and *Polypus tonganus*	*Octopus rugosus (*Bosc, 1792) **taxon inquirendum;** *Abdopus tonganus* (Hoyle, 1885)					✓	Massy, 1916
1920	Arm regeneration	*Octopus vulgaris* (Cuvier, 1797); *Eledone moschata* (Lamarck, 1798); *Sepia officinalis* (Linnaeus, 1758)			✓		✓	Lange, 1920
1929	Arm regeneration *Octopus (Octopus) tonganus*	*Abdopus tonganus* (Hoyle, 1885)					✓	Robson, 1929
1964	Arm regeneration, branchial gland and branchial heart healing	*Octopus vulgaris* (Cuvier, 1797)					✓	Taki, 1964
1977	Arm regeneration	*Sepia officinalis* (Linnaeus, 1758); *Sepiola atlantica* (d'Orbigny [in Férussac & d'Orbigny], 1839–1842); Loliginidae (Lesueur, 1821)			✓	✓		Féral, 1977
1978	Arm and hectocotylus regeneration	*Octopus vulgaris* (Cuvier, 1797)					✓	O'Dor and Wells, 1978)
1978	Arm regeneration	*Sepia officinalis* (Linnaeus, 1758)			✓			Féral, 1978
1979	Arm regeneration	*Sepia officinalis* (Linnaeus, 1758)			✓			Féral, 1979
1981	Tentacle and arm regeneration	*Ommastrephes bartramii* (Lesueur, 1821)				✓		Murata et al., 1981
1985	Arm and tentacle regeneration in *Sepia pharaonis* and *Loligo duvaucelii*	*Sepia pharaonis* Ehrenberg, 1831; *Uroteuthis (Photololigo) duvaucelii* (d'Orbigny [in Férussac & d'Orbigny], 1835)			✓	✓		Nair and Rao, 1985
1992	Arm regeneration in *Octopus digueti*	*Paroctopus digueti* (Perrier & Rochebrune, 1894)					✓	Voight, 1992
2001	Arm autotomy and regeneration in *Octopus (Abdopus) capricornicus* Arm autotomy in *Ameloctopus litoralis, Octopus mutilans*	*Abdopus capricornicus* (Norman & Finn, 2001); *Ameloctopus litoralis* Norman, 1992;*Octopus mutilans* (Taki, 1942)					✓	Norman and Finn, 2001
2003	Arm regeneration and arm-tip light organs regeneration	*Vampyroteuthis infernalis* (Chun, 1903)				✓		Robison et al., 2003
2006	Arm and tentacle regeneration	*Sepia officinalis* (Linnaeus, 1758)			✓			Rohrbach and Schmidtberg, 2006
2006	Arm regeneration	*Wunderpus photogenicus* (Hochberg, Norman & Finn, 2006)					✓	Hochberg et al., 2006
2011	Arm regeneration	*Octopus vulgaris* (Cuvier, 1797)					✓	Florini et al., 2011
2011	Arm regeneration	*Octopus vulgaris* (Cuvier, 1797)					✓	Fossati et al., 2011
2012	Arm autotomy and regeneration	*Octopoteuthis deletron* Young, 1972				✓		Bush, 2012
2013	Involvement of acetylcholinesterase in the arm regeneration	*Octopus vulgaris* (Cuvier, 1797)					✓	Fossati et al., 2013
2014	Arm regeneration	*Sepia officinalis* (Linnaeus, 1758); *Sepia pharaonis* (Ehrenberg, 1831)			✓			Tressler et al., 2014
2015	Acetylcholinesterase expression during adult arm regeneration and embryonic arm development	*Octopus vulgaris* (Cuvier, 1797)					✓	Fossati et al., 2015
2016	Arm regeneration in	*Octopoteuthis nielseni* (Robson, 1948)				✓		Young and Vecchione, 2016
2017	Arm loss and regeneration	*Abdopus* sp. (Norman & Finn, 2001)					✓	Wada, 2017
2018	Arm regeneration (micro-PET imaging)	*Octopus vulgaris* (Cuvier, 1797)					✓	Zullo et al., 2018
**HECTOCOTYLUS REGENERATION**								
1882	Tentacle regeneration in *Ommastrephes illecebrosus;* Hectocotylus regeneration in the family Philonexidae D'Orbigny.	*Illex illecebrosus* (LeSueur, 1821)					✓	Verrill, 1882
1887	Tentacle and hectocotylus regeneration in *Octopus fusiformis, Octopus inconspicuus, Octopus cuvieri*	*Octopus fusiformis* Brock, 1887 **nomen dubium;** *Octopus inconspicuus* Brock, 1887 **taxon inquirendum**; *Callistoctopus lechenaultii* (d'Orbigny [in Férussac & d'Orbigny], 1826)					✓	Brock, 1887
1940	Hectocotylus regeneration in castrated octopus	*Octopus vulgaris* (Cuvier, 1797)					✓	Callan, 1940
1944	Hectocotylus regeneration	*Octopus vulgaris* (Cuvier, 1797)					✓	Taki, 1944
1978	Arm and hectocotylus regeneration	*Octopus vulgaris* (Cuvier, 1797)					✓	O'Dor and Wells, 1978
1995	Hectocotylus regeneration	*Sepietta oweniana* (d'Orbigny [in Ferussac & d'Orbigny], 1839-1841); *Sepiola ligulata* (Naef, 1912)				✓		Bello, 1995
**TENTACLES, ABNORMALITIES**								
2008	Tentacle branching in *Moroteuthis ingens*	*Onykia ingens* (E. A. Smith, 1881)				✓		González and Guerra, 2008
**TENTACLES, AUTOTOMY**								
2012	Tentacle autotomy and regeneration	*Ommastrephes bartramii* (Lesueur, 1821)				✓		Kurosaka et al., 2012
**TENTACLES, REGENERATION**								
1881	Sucker, arm and tentacle regeneration in *Loligo pealeii* and *Ommastrephes illecebrosus*	*Doryteuthis (Amerigo) pealeii* (Lesueur, 1821); *Illex illecebrosus* (LeSueur, 1821)				✓		Verrill, 1881
1882	Tentacle regeneration in *Ommastrephes illecebrosus;* Hectocotylus regeneration in the family Philonexidae D'Orbigny.	*Illex illecebrosus* (LeSueur, 1821)				✓	✓	Verrill, 1882
1887	Tentacle and hectocotylus regeneration in *Octopus fusiformis, Octopus inconspicuus, Octopus cuvieri*	*Octopus fusiformis* Brock, 1887 **nomen dubium;** *Octopus inconspicuus* Brock, 1887 **taxon inquirendum**; *Callistoctopus lechenaultii* (d'Orbigny [in Férussac & d'Orbigny], 1826)					✓	Brock, 1887
1937	Tentacle regeneration	*Sepioteuthis lessoniana* (Férussac [in Lesson], 1831)				✓		Adam, 1937
1966	Tentacular stalk regeneration	*Liocranchia gardineri* (Robson, 1921) **taxon inquirendum**				✓		Clarke, 1966
1968	Tentacle regeneration	*Architeuthis dux* Steenstrup, 1857				✓		Aldrich and Aldrich, 1968
1981	Tentacle and arm regeneration	*Ommastrephes bartramii* (Lesueur, 1821)				✓		Murata et al., 1981
1985	Arm and tentacle regeneration in *Sepia pharaonis* and *Loligo duvaucelii*	*Sepia pharaonis* (Ehrenberg, 1831); *Uroteuthis (Photololigo) duvaucelii* (d'Orbigny [in Férussac & d'Orbigny], 1835)			✓	✓		Nair and Rao, 1985
1996	Tentacle regeneration	*Sepia officinalis* (Linnaeus, 1758)			✓			Hielscher et al., 1996
2006	Arm and tentacle regeneration	*Sepia officinalis* (Linnaeus, 1758)			✓			Rohrbach and Schmidtberg, 2006
2012	Tentacle autotomy and regeneration	*Ommastrephes bartramii* (Lesueur, 1821)				✓		Kurosaka et al., 2012
**NERVE REGENERATION**								
1932	Pallial and stellar nerve degeneration and regeneration in *E. moschata, E. cirrosa, O. vulgaris, O. macropus, S. officinalis, L. vulgaris, Loligo pealeii*	*Eledone moschata* (Lamarck, 1798); *Eledone cirrhosa* (Lamarck, 1798); *Octopus vulgaris* (Cuvier, 1797); *Octopus macropus* (Risso, 1826); *Sepia officinalis* (Linnaeus, 1758); *Loligo vulgaris* (Lamarck, 1798); *Doryteuthis (Amerigo) pealeii* (Lesueur, 1821)			✓	✓	✓	Sereni and Young, 1932
1932	Pallial and stellar nerve degeneration and regeneration	*Eledone moschata* (Lamarck, 1798); *Octopus vulgaris* (Cuvier, 1797); *Octopus macropus* (Risso, 1826); *Loligo vulgaris* (Lamarck, 1798); *Sepia officinalis* (Linnaeus, 1758)			✓	✓	✓	Young, 1932
1972	Pallial nerve and stellar nerve lesion, regeneration and degeneration. Effect of lesion on the stellate ganglion	*Octopus vulgaris* (Cuvier, 1797); *Sepia officinalis* (Linnaeus, 1758)			✓		✓	Young, 1972
1974	Recovery of function after pallial nerve cut or crush	*Octopus vulgaris* (Cuvier, 1797)					✓	Sanders and Young, 1974
2017	Pallial nerve degeneration and regeneration	*Octopus vulgaris* (Cuvier, 1797)					✓	Imperadore et al., 2017
2018	Pallial nerve regeneration (imaging through multiphoton microscopy)	*Octopus vulgaris* (Cuvier, 1797)					✓	Imperadore et al., 2018
**SHELL, REPAIR AND REGENERATION**								
1877	Shell repair in fossil records (Nautiloids) ê	N/A	✓					Barrande, 1877
1964	Shell repair in fossil records (Nautiloids)	N/A	✓					Gordon, 1964
1967	Shell repair in fossil records (Ammonoids)	N/A	✓					Guex, 1967
1972	Shell repair	*Nautilus pompilius* (Linnaeus, 1758)	✓	✓				Haven, 1972
1973	Shell repair in fossil records (Ammonoids)	N/A	✓					Hölder, 1973
1973	Shell repair in fossil records (Ammonoids)	N/A	✓					Saunders, 1973
1974	Shell repair	*Nautilus macromphalus* (G.B. Sowerby II, 1849)		✓				Meenakshi et al., 1974
1975	Shell repair in fossil records (Ammonoids)	N/A	✓					Lehmann, 1975
1976	Shell repair in fossil records (Ammonoids)	N/A	✓					Keupp, 1976
1977	Shell repair in fossil records (Ammonoids)	N/A	✓					Hölder, 1977
1977	Shell repair in fossil records (Ammonoids)	N/A	✓					Keupp, 1977
1978	Shell repair	*Nautilus pompilius* (Linnaeus, 1758)	✓	✓				Tucker and Mapes, 1978
1979	Shell repair in fossil records (Bactritoids)	N/A	✓					Mapes, 1979
1985	Shell, cirri, hood, buccal mass and appendages regeneration	*Nautilus pompilius* (Linnaeus, 1758)		✓				Arnold, 1985
1986	Shell repair in fossil records (Ammonoids)	N/A	✓					Landman and Waage, 1986
1988	Shell repair	*Nautilus pompilius* (Linnaeus, 1758)		✓				Tanabe et al., 1988
1989	Shell repair in fossil records (Ammonoids)	N/A	✓					Bond and Saunders, 1989
1991	Shell repair	*Nautilus* sp. (Linnaeus, 1758)		✓				Saunders et al., 1991
1991	Cuttlebone regeneration in *Sepia officinalis*	*Sepia officinalis* (Linnaeus, 1758)			✓			von Boletzky and Overath, 1991
1993	Shell repair	*Argonauta* sp. (Linnaeus, 1758)					✓	Trego, 1993
1993	Shell repair in fossil records (Ammonoids)	N/A	✓					Kakabadzé and Sharikadzé, 1993
1997	Shell repair in *Nautilus scrobiculatus* and in fossil records (Ammonoids)	*Allonautilus scrobiculatus* (Lightfoot, 1786)	✓	✓				Landman and Lane, 1997
1998	Shell repair in fossil records (Ammonoids)	N/A	✓					Keupp, 1998
2002	Shell repair in fossil records (Ammonoids)	N/A	✓					Morard, 2002
2002	Shell repair in fossil records (Ammonoids)	N/A	✓					Kröger, 2002b
2002	Shell repair in fossil records (Ammonoids)	N/A	✓					Kröger, 2002a
2003	Shell repair in *Nautilus sp*. and in fossil records (Ammonoids)	N/A	✓	✓				Mapes and Chaffin, 2003
2003	Cuttlebone repair in *Sepia orbignyana*	*Sepia orbignyana* Férussac [in d'Orbigny], 1826			✓			Bello and Paparella, 2003
2004	Shell repair in fossil records (Nautiloids)	N/A	✓					Kröger and Keupp, 2004
2004	Shell repair in fossil records (Nautiloids)	N/A	✓					Kröger, 2004
2005	Shell repair in fossil records (Belemnites)	N/A	✓					Mietchen et al., 2005
2006	Shell repair in fossil records (Ammonoids)	N/A	✓					Keupp, 2006
2007	Shell repair in fossil records (Ammonoids, Nautiloids, Bactritoids)	N/A	✓					Klug, 2007
2010	Shell repair	*Nautilus* sp. (Linnaeus, 1758)		✓				Saunders et al., 2010
2010	Shell repair in fossil records (Ammonoids)	N/A	✓					Zato, 2010
2011	Shell repair in fossil records (Ammonoids)	N/A	✓					Slotta et al., 2011
2011	Shell repair in fossil records (Endocerids)	N/A	✓					Kröger, 2011
2012	Shell repair	*Nautilus pompilius* (Linnaeus, 1758)		✓				Tsujino and Shigeta, 2012
2013	Shell repair	*Nautilus pompilius* (Linnaeus, 1758)		✓				Yomogida and Wani, 2013
2013	Shell repair in fossil records (Ammonoids)	N/A	✓					Odunze and Mapes, 2013
2015	Shell repair in fossil records (Ammonoids)	N/A	✓					Hoffmann and Keupp, 2015
**OTHER**								
1881	Sucker, arm and tentacle regeneration in *Loligo pealeii* and *Ommastrephes illecebrosus*	*Doryteuthis (Amerigo) pealeii* (Lesueur, 1821); *Illex illecebrosus* (LeSueur, 1821)				✓		Verrill, 1881
1933	Sucker regeneration	*Octopus vulgaris* (Cuvier, 1797)					✓	May, 1933
1964	Branchial gland and branchial heart healing	*Octopus vulgaris* (Cuvier, 1797)					✓	Taki, 1964
1981	Cornea regeneration in *Octopus dofleini* and *O. vulgaris*	*Enteroctopus dofleini* (Wülker, 1910); *Octopus vulgaris* (Cuvier, 1797)					✓	Dingerkus and Santoro, 1981
1985	Shell, cirri, hood, buccal mass and appendages regeneration	*Nautilus pompilius* (Linnaeus, 1758)		✓				Arnold, 1985
2000	Muscle repair in fossil records (Ammonoids)	N/A	✓					Keupp, 2000
2003	Arm regeneration and arm-tip light organs regeneration	*Vampyroteuthis infernalis* (Chun, 1903)					✓	Robison et al., 2003
2008	Jaw repair	*Nautilus belauensis* (Saunders, 1981); *Nautilus macromphalus* (G.B. Sowerby II, 1849); *Nautilus pompilius* (Linnaeus, 1758); *Allonautilus scrobiculatus* (Lightfoot, 1786)		✓				Kruta and Landman, 2008
2011	Chromatophore re-growth during fin regeneration	*Sepia officinalis* (Linnaeus, 1758)			✓			Yacob et al., 2011
2017	Muscle regenerative potential in cephalopods	N/A			✓	✓	✓	Zullo et al., 2017

*A total of 119 studies are included in this list organized by topic (e.g., wound healing; arm abnormalities, autotomy and regeneration; hectocotylus regeneration; tentacle autotomy and regeneration; nerve regeneration; shell repair and regeneration) and chronological order. For each study, we provide a general description based on the topic and indicate the taxon (including fossil record) and the species that has been subject of the work. The taxonomy has been revised following WoRMS (World Register of Marine Species, http://www.marinespecies.org/index.php) whenever the case, and reported as in the original study (General Description) when the species differ from the currently accepted taxonomic nomenclature. In a few cases some species are indicated as nomen dubium (a species name is of uncertain taxonomic significance, no type and original description very vague) and taxon inquirendum (when the taxonomic validity is uncertain or disputed by different experts). In the table we do not include the work by Young on the anatomy of the nervous system of Octopus vulgaris, that provide description of regeneration occurring in the “central nervous system” in various regions of cephalopods' brain. N/A, not available*.

## Wound healing

Skin, fin and arm damage occurs frequently in the course of a cephalopod lifespan as a result of such events as predator-prey interactions, agonistic and reproductive encounters, capture and transportation, and autotomy during predator evasion and autophagy (e.g., Hanlon et al., 1984; Budelmann, 1998; Florini et al., 2011; Bush, 2012). After injury, damaged structures can heal and recover their function, although wound repair appears delayed in fully mature animals, often leading to the failure of skin lesions to heal (O'Dor and Wells, 1978).

### Cephalopod skin and wound healing

The skin of cephalopods plays an important role in (i) concealment and communication and as (ii) a barrier that protects the animal body (review in e.g., Packard, 1988). Polglase and his colleagues were the first to describe the process of wound healing following injury to the skin of the mantle (*Eledone cirrhosa*, Polglase et al., 1983). Soon after surgery, octopuses (kept at 10–11°C) were seen holding and rubbing the wound with an arm tip. In the first 12 h following injury, in-folding of the epidermis close to the wound due to muscular contraction was observed (Polglase et al., 1983).

Within an hour, the wound surface appears to be covered by dense amorphous eosinophilic material containing necrotic fibroblasts, which increase in number 3 h after lesioning. At this time, contraction of the adjacent skin continues, significantly reducing the size of the wound. About 5 h after injury, hemocytes proliferate at the site of the wound through diapedesis, accelerating at about 12 h post-lesion. This acceleration co-occurs with swelling of the central area of the wound, which is also exacerbated by migration of epidermal cells to the wound site (Polglase et al., 1983).

The following day, hemocytes penetrate deeper into the wound and transform from their classical round shape to a fusiform one. These cells eventually cover the entire wound, forming a dermal plug at about 30 h post-lesion, aided by inward migration of the epidermis surrounding the injury. This epidermal migration, which becomes extremely evident at 2 days post-lesion, is made possible by penetration of cells through the hemocyte plug (Polglase et al., 1983).

An increase in cellular organization is then observed at 3–4 days post injury. Notably, hemocytes assume the fibroblast cell type appearance. During this period, the size of the wound continues to shrink, with the closure usually completed after 5 days. Return to the normal morphology of the epidermis, however, was only achieved at least 50 days post-lesion, and slow, continuous contraction of the wound occurs at up to 150 days post-lesion (Polglase et al., 1983).

The existence of fatal ulcerative lesions in some laboratory-reared octopus species (Polglase, 1980; Hanlon et al., 1984) has led scientists to question the efficacy of the healing process in the presence of pathogens (Bullock et al., 1987). Bacterial infection appears to inhibit muscular contractions of the wound at early stages, as well as induce a greater response in hemocytes.

Normally, hemocytes are actively involved in the removal of necrotic tissue from the wound and in the formation of additional amorphous layers (usually one or two) that create supplementary barriers to protect healthy tissue. However, when pathogens are present, these blood cells often appear to be necrotic and to exhibit cytoplasmic granulation, especially when they are in close proximity to bacteria. The observed cell necrosis is thought to be induced by toxins produced by the pathogens. Even when bacterial activity at the wound site is no longer observable a few days after injury, epidermal migration can still be delayed, resulting in incomplete closure of the wound up to 7 days later.

### Wound healing of appendages after amputation

The aforementioned process of wound repair also characterizes the first phases of regeneration after arm damage (see for example studies in: Lange, 1920; Féral, 1977, 1978, 1979, 1988; Fiorito et al., 2014; Zullo et al., 2017) and determines the course of repair that follows (Féral, 1988). This process was first reported by Lange (1920) in several species, and then several years later by Féral (1978, 1979, 1988) in *Sepia officinalis*. More recently, Shaw et al. (2016) described the process as it occurs in *Octopus vulgaris*.

These studies have identified several variables that affect the speed of healing, including temperature, relative position of the injury (i.e., distal portion of the arm versus proximal), species, animal age, body size, and health status of an individual, among others.

Although several studies have demonstrated that the healing of a damaged arm requires at least 24 h, the timing is highly variable; some wounds may show little or no healing even after 30 h (Lange, 1920). Complete healing of an arm in *S. officinalis* requires about 5 days at temperatures between 14 and 19°C, and up to 2 weeks at 10°C (Féral, 1988). Interestingly Shaw et al. (2016) found that time of healing might also depend on “innate” differences in self-regenerative capacity. In comparing two sub-populations of *O. vulgaris*, one was found to heal significantly faster than the other. Six-hours after arm injury, the “fast” healers exhibited 80% coverage of their wound, while only 50–60% coverage was noted in the second group of animals.

Lange observed that immediately after a lesion to an arm, no bleeding occurs (1920). The edges of the wound, consisting mainly of dermal connective tissue, begin contracting around the lesion, as occurs in damage to skin on other parts of the body (Polglase et al., 1983). Only the most external part of the wound is covered, leaving the central area of the injury exposed and the axial nerve protruding from the wound in the most severe cases. Transverse muscle degeneration is also evident soon after injury.

A few hours after lesion (ca. 5 or 6 h), blood enters and covers the wound, forming a blood clot which is later resorbed. Blood corpuscles also rush to the lesion and transform from the spherical circulating-type to a spindle shape. They also appear to undergo division, as the total number present at the wound site increases with time (Lange, 1920), although no mitosis is detected, suggesting that proliferation is occurring through direct or amitotic division (see below). These cells form cicatricial tissue, which creates an initial barrier to the external environment.

Later, the epithelium begins to regenerate through morphallaxis as old material rearranges itself, covering the cicatricial tissue, which is retained underneath. This structure, called the “primary blastema,” is thought to be involved in supplying material for the regenerating connective tissue. Epithelial cells, after covering the entire wound, then change their shape from flat to cubic and initiate nuclear (and possibly amitotic) division (Lange, 1920).

In subsequent studies, Féral (1988) investigated the role of two types of fibrous material in the wound healing process of *S. officinalis*. A first type was identified as covering the nerve cord and muscles and forming a network between amoebocytes (i.e., hemocytes) in the scar tissue. A second type, made of collagen fibers, appears in the hypodermis. Agglutinated amoebocytes form scar tissue which is eventually infiltrated by collagen fibers that reinforce the scar and are probably produced by the blastema. A maximum amount of collagen is reported at 48 h after amputation, followed by a decrease to the initial levels at the end of the cicatrization phase. However, this process varies depending on temperature.

Almost a century after the first study by Lange, Shaw et al. (2016) investigated the process of regeneration in *O. vulgaris*. These authors suggest that muscle cells also contribute to the formation of the plug covering the wound, as well as the previously-described actions of hemocytes.

Along with the constructive processes initiated by hemocytes and muscle cells, destructive processes (i.e., cell death) of damaged tissues is also observed in the skin, muscles and nerve cells within the first 6 h after injury.

## Regeneration of cephalopod body parts

### Appendages

Cephalopod appendages (i.e., arms and tentacles) are extremely flexible muscular hydrostats lacking fluid-filled cavities (a hydrostatic skeleton is characteristic of many other invertebrates) and hard skeletal supports (review in: Kier and Smith, 1985; Kier, 2016). Each arm is composed of a nerve cord running along the central axis of the appendage, surrounded by three muscle bundles (transverse, longitudinal and oblique) each perpendicular to each other (see description in Margheri et al., 2011).

Arm damage seems to be a common occurrence among cephalopods in the wild (e.g., Steenstrup, 1856; Brock, 1887; Bush, 2006, 2012). For example, Florini et al. (2011) found that 51% of *O. vulgaris* collected from fishermen in the Bay of Naples (Italy) showed damage to one or more arms; Voight (1992) observed similar degrees of damage in 26% of *O. digueti* (from Cholla Bay, Mexico). In both species, dorsal arms appeared to be more affected than ventral ones. It is also notable that in *Abdopus* sp., where sneaker mating tactics are observed among small males, the frequency of arm loss in sneaker males was found to be 100%, compared to 25% in the males mate-guarding a female (population mean = 37%; Wada, 2017).

Although the ability of cephalopods to survive arm and tentacle loss has been known since antiquity (see accounts in *Historia Animalium*; Aristotle, 1910), the first paper formally describing arm regeneration in cephalopods dates back to the mid nineteenth century, when Steenstrup described the main structural features of the arms, including “sexual” appendages and their specialization (i.e., hectocotylus) in some cephalopod species, and focused in particular on the ability of octopods to regenerate arms lost during copulation, injured or bitten off by predators (Steenstrup, 1856, 1857). Streenstrup considered decapods (cuttlefish and squid) to be incapable of re-growing lost appendages, maintaining only a capacity for wound healing. This was confirmed in a later study (Brock, 1887).

Nevertheless, decapods are not completely lacking in regenerative ability; Verrill (1881) observed regenerating suckers in some species of squid (e.g., *Loligo pealei* and *Ommastrephes illecebrosus*; see Table 1).

The nineteenth century was characterized by the discovery of many new cephalopod species, a large proportion of which were found to possess regenerative abilities (Verrill, 1881; Brock, 1887; Riggenbach, 1901), including abnormalities such as “arm dichotomy,” i.e., bifurcation (Appellof, 1893; Parona, 1900; Hanko, 1913). Most accounts were merely descriptive, lacking any experimental investigation.

**Figure 3:**
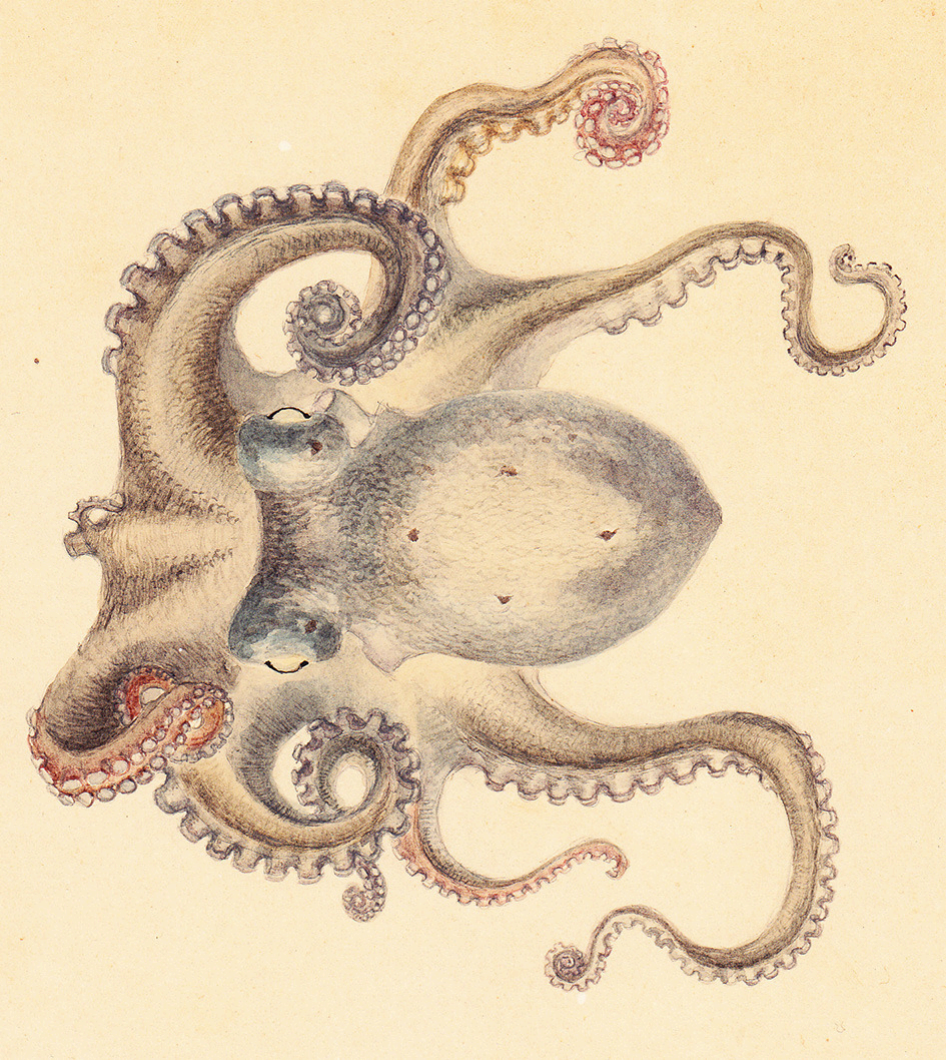
Regenerating arm in octopus. A scientific drawing of (possibly) *Octopus vulgaris* showing the first right arm regenerating after a lesion that occurred in the most proximal part of the arm. The stump shows a big sucker and a regenerating tip. The third left arm shows the apparent effect of an injury, as it is much shorter and thinner than the other arms. Drawing is a gift to the Association for Cephalopod Research - CephRes from a private collection.

At the beginning of the twentieth century, Lange initiated a detailed investigation of arm regeneration in three cephalopod species—*S. officinalis, O. vulgaris*, and *Eledone moschata*—employing both macroscopic observations and histological analysis (Lange, 1920). Her work was based on specimens kept at the Stazione Zoologica (Naples, Italy) as well as at Musee Oceanographie (Monaco) and inspired and guided by Carl Chun and Johann Georg Grimpe, who also provided guidance on the standardization of animal care (Grimpe, 1928). At that time at the Stazione Zoologica, the classical scientific illustration provided examples of the phenomenon originally described by Riggenbach (1901; see also Figure 3) that clearly inspired Lange's study.

Lange's work divided the process of arm regeneration into three stages: wound healing (previously described, see above), tissue degeneration and renewal. Her study also established that: (i) the whole process of arm regeneration occurs through morphallaxis, i.e., existing tissues are rearranged and then regenerated into new tissues (except for dermal connective tissue); (ii) cell proliferation seems to occur through amitotic division, since mitosis was never observed; (iii) cuttlefish are indeed capable of regenerating lost appendages, contrary to Steenstrup's earlier assertion that decapods lack regenerative ability, but this is thought to occur via “compensatory regulation,” i.e., development of a rudimentary buccal arm rather than actual regeneration of the lesioned arm; (iv) the arm tip, which Lange considered to be made of tissue at an undifferentiated embryonic stage, requires less time to regenerate and form the embryonic blastema than that required if the lesion is made at the base of an arm, where tissues are more differentiated (Lange, 1920).

Immediately after an arm lesion, muscles close to the wound begin degenerating, with the sarcoplasm breaking down and the spiral fibers apparently growing thicker. As degeneration advances, fibers begin losing their cylindrical shape becoming a “clotty mass.” During this process, muscle nuclei change shape, becoming round and later fragmenting into two or three particles.

These fragments are then absorbed by the corpuscles that migrate from the blood clot to the muscles. Muscle regeneration is characterized by the appearance of large cells containing little protoplasm and one large nucleus. These are likely to be sarcoblasts originating from the area where muscles tissue has degenerated. In Lange's view, sarcoblasts are the only possible source of muscle fibers (Lange, 1920). Later, they move to the most distal part of the wound and collaborate with neuroblasts in the formation of the second blastema, increasing their number by mitosis.

Twelve to fourteen days are required for sarcoblasts to differentiate into muscle fibers, with the longitudinal ones being the first to begin this process close to the perimuscular connective-tissue membrane. Transverse muscles seem to require more time. Proliferation of the central muscle bundle dictates the production of sucker muscles, which also involves sarcoblasts, this time arranged in two parallel layers around the cavity of the forming sucker, and later developing into radiating and circular muscles.

Degeneration of the nerve cord also begins soon after lesioning and proceeds quite quickly, starting from the nuclei of the layer of ganglion cells. Waste from the nucleus usually disappears quickly, but some persists. Degeneration also involves glial cells whose nuclei shrink while fibers of the myelin cord swell, with degeneration being more marked and pronounced than in the ganglia layer and neuropil.

Around 10 h after surgery, the number of nuclei increases in the neuropil and in the myelin cord due to the migration of blood corpuscles and amitotic division of the glia nuclei. One or two days after lesioning, neuroblasts appear in the neuropil, later migrating to the distal part of the stump to form the second blastema. The source of these neuroblasts is thought to be either glial cells or small nerve cells (Lange, 1920).

Next, well-differentiated fibers of the myelin cords grow into the second blastema separating neuroblasts from sarcoblasts, producing neuropil fibers.

More time is required for neuroblasts to form ganglion cells, protoplasm, and fibers. An axial nerve requires 3 weeks to fully develop, while large ganglia probably appear very late. The axial nerve tends to occupy the majority of the regenerating stump, while in a normal arm, it occupies a quarter of the total volume.

Lange was not able to identify sucker ganglia or the four nerve cords in the muscles of the regenerating tissue, nor was any data on the regeneration of the vascular system available at the time of the study (Lange, 1920).

From a macroscopic point of view, Lange (1920) highlighted the involvement of the two suckers closest to the lesion. Soon after lesioning, they assume an abnormal position which helps in closing the wound. This position is retained for at least 2 or 3 days, and even up to some weeks before the suckers return to their initial location. When this occurs, a little knob is observed near the external part of the regenerated portion of the arm, while in the interior of the knob, a groove is formed. It is from this groove that suckers later regenerate, initially in a single row (all species), and later in paired rows (*O. vulgaris*), though one sucker remains unpaired. Though sucker re-innervation during arm regeneration was not observed by Lange (1920), May (1933) demonstrated through histological analysis that newly forming suckers attract nervous fibers from the central nervous axis, supporting Cajal's neurotropic theory.

The majority of reports regarding the regeneration of cephalopod appendages have been based on octopods, while published data on decapods remains scarce. Lange (1920) attributed this to both an overall lack of knowledge and the great difficulties associated with, rearing decapods compared to octopods (see accounts in, Lange, 1920; Sereni and Young, 1932; Taki, 1941), as well as a reduced frequency of arm and tentacle mutilation in squids and cuttlefishes (Lange, 1920; Adam, 1937). However, these and other assumptions by of Lange were questioned by Aldrich and Aldrich (1968) who investigated, again macroscopically, a specimen of the giant squid *Architeuthis dux* undergoing tentacle regeneration. They also discussed previous data on the frequency of regenerative phenomena occurring in decapods (at least in *Loligo pealei, Illex illecebrosus, A. dux*, and *Architeuthis harveyi*) which suggested that Lange underestimated the phenomenon (1920). While not completely refuting the hypothesis of “compensatory regulation,” the authors go so far as to suggest that Lange's theory might have stemmed from a misinterpretation of arm or tentacle dimorphism (Aldrich and Aldrich, 1968).

It was only at the end of the 1970s, with improvements in breeding conditions for *S. officinalis*, that Jean-Pierre Féral was able to perform detailed studies of the process of arm regeneration in this species. Complete arm regeneration and functional recovery was achieved after 2–3 months (at 16°C) following experimental lesion to young cuttlefish. Regenerative capacity was dependent on age, physiological state and water temperature, with adults exhibiting diminished or no regenerative capacity after wound healing during late autumn or winter, particularly when seawater temperatures dropped below 14°C (Féral, 1978, 1979).

**Figure 4:**
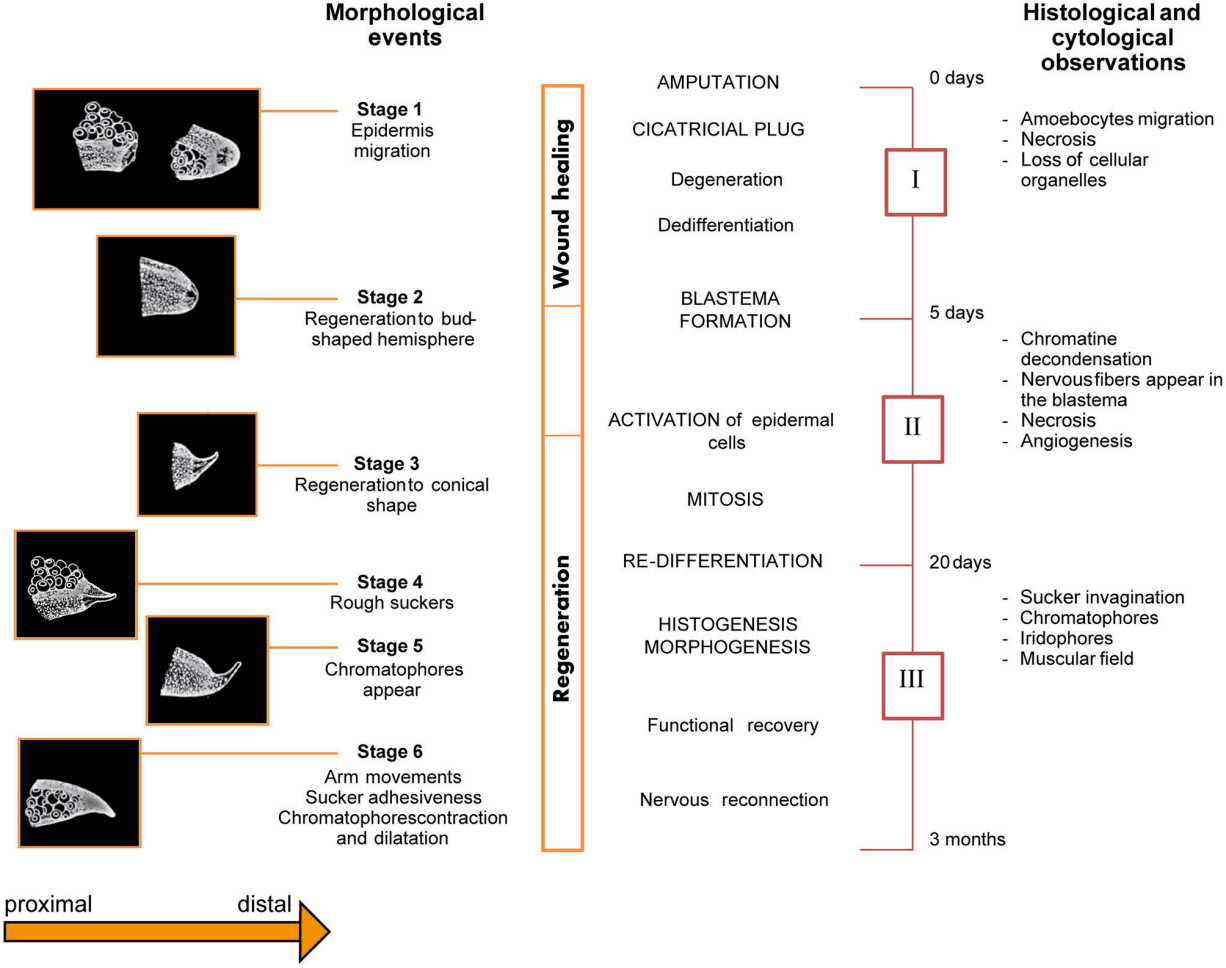
Schematic overview of the series of events occurring during arm regeneration in the cuttlefish. Stages (Left) and phases (Right) of regeneration occurring after arm damage in *Sepia officinalis* are depicted as originally described by Féral. The diagram presents an overview of the morphological (six stages), histological and cytological (three phases) events occurring during arm regeneration. Adapted from Féral (1978, 1979) after permission of CBM - Cahiers de Biologie Marine.

Féral identified six stages of regeneration by morphology (Figure 4) based on histological and cytological analyses (Féral, 1978; 1979). Those findings largely concur with Lange's observations of octopus arm regeneration. The six stages are summarized below:

***Stage 1** (from surgery to day-7)*: characterized by the protrusion of the central nervous axis and contraction of the wound's edge. A few hours after lesioning, one or two suckers adjacent to the lesion move forward; they assume their normal position only 2 or 3 days later. Five to seven days are required for the epidermis to completely cover the wound.
***Stage 2** (day 5 to 14)*: due to swelling of the scar at the level of the nervous axis, a bud-shaped hemisphere appears at the injury site.
***Stage 3** (day 10 to 21)*: characterized by the development of the regenerating tissue into a conical shape.
***Stage 4** (day 17 to 25)*: rough suckers appear first on the ventral side of the stump closest to the lesion and then on the regenerating tissue.
***Stage 5** (day 25 to 35)*: chromatophores gradually appear within the regenerating tissue.
***Stage 6** (beyond day 30)*: The regenerated arm regains its functionality. It becomes thicker, the new suckers gain function, and chromatophores increase in number, growing larger and darker.

Based on the histological and cytological events occurring during arm regeneration in the cuttlefish, the following three phases were identified by Féral (1978, 1979); see Figure 4:

**Phase I** (corresponding to Stage 1, above): characterized by wound healing, degeneration of nerve cords, muscles, and blood vessels, as well as cell de-differentiation.
**Phase II** (corresponding to the end of Stage 1, Stage 2, and part of Stage 3; from day 5 to 20): represents the starting point of regeneration, during which blastema formation, cellular activation and growth of the regenerating tissue occurs.

The blastema is composed of de-differentiated cells which increase in number during the first 10 days, though no mitotic event is evident. At a certain point, cells activate and begin changing their appearance. Growth of the regenerating tissue starts at this point. Nervous fibers infiltrate the blastema and mitosis starts at the end of the second week.

The brachial artery penetrates the blastema along with the axial nerve cord. The epidermis appears to be multilayered, but by the end of the third week, it is again composed of a single layer.

**Phase III** (corresponding to the end of Stage 3, and to stages 4, 5 and recovery of function: from day 20 to the third month): in this phase, the arm is observed to re-grow and cell differentiation occurs. Indeed, after the third week, mitotic events appear to wane and cells begin to differentiate in a concentric field around the nerve cord. The nervous system is the first tissue to differentiate: extending fibers of the cerebro-brachial tract penetrate into the blastema; later, the ganglionic layer formed by dividing neuroblasts appears. During this phase, putative glial cells support nerve fibers during regeneration. At around day 20, they proliferate and follow the axons' progress.

The axial nervous system, the brachial artery, and “epineuraux” (Féral, 1979) muscles differentiate jointly. Intrinsic longitudinal muscles become visible on the 20th day, together with the collagen that protects them from the outside environment. Later, extrinsic longitudinal muscles appear, followed by transverse muscles. Development of the longitudinal muscle bundles occurs through alignment of fusiform myoblasts along belts parallel to the nervous axis. Muscle cells differentiate in muscular fields of the stump. Myofilaments appear immediately and grow slowly between longitudinal muscles and nervous cord. At the beginning of the differentiation phase, while the transverse muscles are limited in thickness and built of myoblasts, the longitudinal muscle cells rely on the presence of myofilaments. During the second month post-lesion, the transverse muscle increase in thickness, with mitotic activity still visible (Féral, 1979).

During the third week, proliferation of the central fascicles induces the formation of sucker (or acetabular) muscles. During invagination of the sucker chambers, muscle cells first form one and then several parallel layers. These cells become the radial muscles and sphincters. Subsequently, acetabulo-branchial muscles also differentiate. Suckers innervation occurs only at later stages, when the suckers are completely formed (at around the 40th day), and they become functional only 3 months after injury.

Chromatophores are identifiable among the fibroblasts before they actually appear on the skin due to the presence of pigmented grains. At around day 20, the cells of the dermis differentiate, apparently originating from hemocytes. Iridophores appear some days later (day 25 to−27) and initially, they are positioned without a specific orientation. Later, they arrange themselves in parallel to each other.

The radial muscles of the chromatophores differentiate when the muscles form; however, their innervation occurs later. Indeed, fibers from the median nervous axis start growing at the end of the third week, even though the complete innervation of chromatophores and iridophores does not occur until between the second and third month after lesioning. The basal membrane of the epithelial cells appears at the moment of differentiation; it folds to form the initial structure of the suckers and then invaginates to form the suction and adherent chambers. This occurs along with the penetration of the brachial vein into the regenerating tissue (Féral, 1979).

Amoebocytes are the only cells that travel to the lesion from other parts of the body. However, when this migration stops, the number of cells forming the blastema continues to increase, despite the fact that no mitotic events can be observed. Instead, this appears to be due to local cellular reorganization. Within the lesion, damaged cells degenerate and are removed, while others de-differentiate, losing their particular features and becoming a source of regenerative cells.

After de-differentiation, muscle and nervous cells appear to be capable only of differentiating into the original cell type, whereas connective tissue cells may originate from either fibrocytes or amoebocytes (Féral, 1979).

Féral compared his results with Lange's observations and proposed that the same stages occur in all three species examined, i.e., *S. officinalis, Sepiola atlantica* and *O. vulgaris*. Specifically wound healing corresponds to **stage 1**; blastema formation and early growth **to stage 2**; later growth to **stage 3**; differentiation and morphogenesis **to stage 4 and 5**; and functional recovery **to stage 6** (Lange, 1920; Féral, 1977, 1979).

At the beginning of this century, interest in the ability of cephalopods to regenerate appendages has been rekindled (e.g., Rohrbach and Schmidtberg, 2006; Fossati et al., 2011, 2013, 2015; Tressler et al., 2014; Imperadore et al., 2017; Zullo et al., 2017). Recent studies largely confirm with the results obtained by Lange and Féral, albeit with some differences, particularly with regard to the timing of each stage.

Tentacle regeneration in *S. officinalis* has been shown to proceed via the same six stages as arm regeneration in the same species, although with a delay in sucker regrowth. In this instance, sucker regeneration in adults appears to proceed through the same steps of sucker formation as cuttlefish embryos, with the process again delayed by comparison (Rohrbach and Schmidtberg, 2006).

A similar process was also proposed for *O. vulgaris* during the study of arm development in embryos (Nödl et al., 2015). Apparently, both development and regeneration of the arm involve similar steps, including “a shift from an early isotropic, mesenchymal cell proliferation to a distally regionalized cell division pattern, as well as the formation of suckers as a single row of rounded papillae” (Nödl et al., 2015, p. 14).

Impairment of function after arm amputation in cuttlefish (*S. officinalis* and *Sepia pharaonis*) has only been reported by Tressler et al. (2014). Indeed, soon after an arm is injured, the motions associated with swimming, prey manipulation and posture are altered. Recovery of function occurs a few days later, long before complete regeneration of the arm, which is reported to require about 40 days. The stages of regeneration appear to be similar to those reported by Lange and Féral, with some differences in the length of each stage. This, as well as other variations in the timing of regeneration stages observed in these studies could be attributed to several factors, including differences in animal age, diet, rearing conditions, water temperature, surgical procedures or anesthesia employed.

Fossati et al. (2013, 2015) describe the morphological changes involved in arm regeneration in *O. vulgaris*, with a focus on the involvement of the enzyme acetylcholinesterase (AchE). The authors found that AchE expression decreases during wound healing, when proliferation activity is intense and rises again above basal level at 3–4 weeks post-lesion. Another decrease is observed 42 days after damage, with a return to basal level 130 days later, when all structures have been regenerated. AchE appears to have a similar expression pattern during regeneration and arm development, suggesting the involvement of this enzyme in functions other than classical synaptic transmission, such as tissue morphogenesis (Fossati et al., 2013, 2015).

### Regeneration of the male cephalopod's “sexual” arm

The hectocotylus is the differentiated-specialized extremity of the “sexual” arm of a male cephalopod. This structure was studied by Sereni (1929a, 1932) who investigated the possibility that a sex hormone controls regeneration of this specialized arm. To answer this question, specimens of *O. vulgaris* were castrated and then had either the hectocotylus tip (males) or the corresponding arm tip (females) removed (Callan, 1940). Complete regeneration of the original structures was observed in both sexes suggesting that the regeneration of both sexual and non-sexual arms do not rely on hormone secretions of the reproductive system. These findings were later confirmed by Taki (1944).

Regeneration of the “sexual arm” was also investigated in later studies.

For example, O'Dor and Wells (1978), induced gonadotropin release by the optic gland, thus forcing sexual maturation of *O. vulgaris* individuals, after which arm-cropping was performed. It was found that in general, faster-maturing octopus of both sexes regenerate their arms more slowly than control animals and, more importantly, that hectocotylized arms regenerate faster than the other arms on the same animal.

In addition, the hectocotylus seems to be less susceptible to injury in the first place in comparison to other arms (Steenstrup, 1857; Bello, 1995). Indeed, some cephalopod species are known to hold this arm close to the body while foraging, presumably to reduce the chances of injury. More rapid regeneration and protection of this specialized arm appear to be due to its importance in mating and reproduction (Huffard et al., 2008). There is even a striking case of a specimen of *Abdopus* sp. which had lost all its arms except the hectocotylus (Wada, 2017).

### Regeneration events in the cephalopod central nervous system

Information regarding the ability of cephalopods to regenerate central nervous tissue is provided by the definitive work of John Z. Young and his co-workers (summarized in Young, 1971). Many experiments involving the removal or lesioning of specific areas of the brain of *O. vulgaris* were carried out with the aim of evaluating subsequent impairment in learning capabilities. In reporting these experiments, Young described the formation of scar tissue above the surface of the brain after removal of a specific brain lobe. He also identified regenerating nerve fibers 34 days after surgery. According to Young, some of these fibers originate from the optic tract, while others from other areas such as the cerebral tract and the palliovisceral system. Regenerating nerve fibers were also identified four days after removal of the subvertical lobe and 16–29 days after bilateral section of the optic tracts.

The distances traveled by the regenerating fibers in the central nervous system of the octopus seem quite remarkable, and further investigation is required to confirm and better describe the phenomenon of neural rewiring. To the best of our knowledge, Young's are, unfortunately, the only available accounts of nerve fiber regeneration in the central nervous system of cephalopods.

### Pallial and stellar nerves

Fredericq (1878) first discovered and described the “phenotypic” effect of transecting one of the two pallial nerves while studying *O. vulgaris* physiology. This pair of nerves connects the brain to the periphery (i.e., the mantle) through the stellate ganglia. Each ganglion gives rise to 25–40 stellar nerves which innervate chromatophores and respiratory muscles in the mantle. Fredericq observed complete paralysis of these muscles and paling of the skin due to the effect of denervation of chromatophores on the mantle, ipsilateral to the lesion. Lesioning of both nerves led to the death of the animal, due to blockage of respiratory movements (Fredericq, 1878).

Many years later, Sereni (1929b) and Young (1929) conducted a series of systematic observations of the consequences of the transecting the pallial and stellar nerves in *O. vulgaris, Octopus macropus, and E. moschata*, as well as the removal of the entire stellate ganglion. After transection of both pallial and stellar nerves, degeneration of nervous fibers and accumulation of lipid material in the nerve stumps was observed. In addition, clot formation occurred between the two stumps of the lesioned nerve (Young, 1929).

After lesioning of the pallial nerve, structural changes were observed in the cells of the subesophageal mass of the brain, where the majority of the fibers originate, but never in the stellate ganglion. Transection of the stellar nerves demonstrated, instead structural changes of the cells inside the ganglion. No signs of regeneration or restoration of function were detected (Young, 1929). Aside from providing a basis for subsequent and more detailed investigations of regeneration, these studies allowed an initial interpretation of the neural pathways and connections between central and peripheral nervous systems via the pallial nerve in cephalopods.

The proof that these nerves are actually able to regenerate was obtained only in 1932, when more than 200 animals representing seven different cephalopod species (both decapods and octopods) were surveyed in an in-depth investigation of the degenerative and regenerative phenomena occurring after pallial and stellar nerve lesioning (Sereni and Young, 1932; Young, 1932). One of the main findings was that scar tissue, mainly produced by amoebocytes, forms between the transected ends of a nerve, and these cells also infiltrate the two stumps and proliferate amitotically. They have also been observed to actively phagocytose and become filled with granules of fat.

Degeneration of axons is mainly observed in the peripheral stump, which breaks into lumps, whereas closer to the lesion, tip ends swell and later branch. Breaking axons produce large spheres which are probably invaded by amoebocytes and which persist even after functional regeneration occurs. Degenerating spheres are also observed after double sectioning of the pallial nerve on both ends of the isolated nerve portion. Regeneration is visible in the few intact fibers of the peripheral stump, though it is much more evident in the central stump, with a calculated growth rate of between 7 and 18 μm per hour. Fibers are able to grow either through the scar, toward the peripheral stump, or laterally and backwards, without a well-defined direction. From 11 to 18 days post lesion, vigorous regeneration of the peripheral stump is also observed. While this is occurring, connective tissue becomes highly disorganized (mainly in the peripheral stump) with nuclei undergoing changes in shape close to the lesion (Sereni and Young, 1932).

Regarding the effect of lesions on the stellate ganglion, it was observed that retrograde degeneration occurs in ganglion cells if the lesion is performed on stellar nerves, while no effect is visible in these cells if the lesion is performed at the level of the pallial nerve; degeneration of the nerve fibers never seems to extend beyond a synapse (Young, 1932). However, transection of the pallial nerve does result in the filling of the ventral neuropil of the ganglion with fine granules which disappear in about 4–5 days. Degeneration is also observed inside the neuropil and in the dorsal roots of the stellar nerves (probably comprising chromatophore fibers, which do not form synapses in the ganglion). At 7 days post-lesion, the neuropil shrinks, resulting in a reduction in the size of the stellate ganglion. After a stellar nerve lesion, no degeneration of the ventral neuropil occurs, though some takes place in the fibers of the dorsal neuropil of certain axons in the pallial nerve (Young, 1972).

Regenerative and degenerative processes appear to correlate strongly with seawater temperature; the speed of both processes has been observed to increase at higher temperatures (Sereni and Young, 1932; Young, 1972).

During these studies, observations of the behavioral effects of lesions to the skin were also carried out. At first, chromatophore muscles are relaxed and thus appear pale, but then they gradually re-expand, showing the ability to re-establish coloration of the skin again 3–5 days after denervation, in a manner independent of the central nervous system (Sereni, 1929b). A “wave effect” is also sometimes observed; this is due to the hyperexcitability of chromatophores (Sereni, 1929b; Sereni and Young, 1932). This phenomenon was described in greater detail by Packard (1992) who named these waves “wandering clouds,” as they propagate randomly over the denervated skin and can last for weeks or even months.

Sereni and Young (1932) observed the first signs of true functional regeneration 65 days post-lesioning, though the majority of the animals required 3–4 months for complete recovery.

Young and his co-workers later focused on the ability of *O. vulgaris* to regain lost function after crushing or cutting one of the pallial nerves (Sanders and Young, 1974). The return of control of color patterning, papillae and mantle muscle contraction was observed over 126 days after surgery by tracking a specific chromatic pattern, the “conflict mottle” (see definition of “Broad Conflict Mottle” as reviewed in Borrelli et al., 2006). This was elicited by placing an animal in a conflict situation, using for example a 10 V shock each time the animal tried to attack a crab prompting uncertainty as to whether to of attack or desist. Eight to ten weeks were required for the complete recovery of pattern production after crushing of the nerve. No animal showed any signs of color pattern recovery until at least 50 days after surgery, in both summer and autumn. Six out of 10 animals recovered the full color pattern (most between 60 and 69 days), while only two out of 10 recovered papillae function (between 30 and 50 days).

When the pallial nerve was cut, only four in 10 animals recovered color patterning, and for these, although some signs of recovery where visible at 30 days, a complete recovery of function required 109 days. By contrast, seven out of 10 animals recovered the ability to raise their papillae. In two of these animals, a 2 cm portion of the nerve was removed during surgery. The skin did not undergo any color changes during the 109 days post-surgery, with chromatophores remaining hyper-excitable and dark spots appearing at random.

Electrical stimulation demonstrated that in these two cases no functional regeneration occurred, while stimulation of the cut pallial nerve after 126 days yielded mantle muscle contractions in three out of three instances, and chromatophore contraction in two out of three instances (in the third instance only a partial response was elicited). Histological analysis of the samples showed pronounced differences in the response of fibers to crushing versus cutting. In the former instance, degenerating axoplasm is confined to the connective tubes and remains visible for months. Fibers were seen to grow in a much more regular fashion compared to crisscrossing of fibers in the cut nerve, despite the fact that in some cases the peripheral stump had been reached. Strikingly, stump-reconnection after cutting often did not lead to functional recovery whereas after crushing it often did. An explanation that has been posited for functional recovery after resection is that the nerve fibers reconnect with their target end-organs. However, the possibility that each individual fiber could both recognize its own specific tube and innervate its original target organ seems quite remote. An alternative possibility is that one axon innervates all the chromatophores of a particular component of the body pattern, rather than just one or a few chromatophores.

Recently, cell proliferation after pallial nerve transection was investigated by Imperadore et al. (2017), who described the mitotic division of circulating hemocytes which migrate to the injury site and continue to proliferate even after infiltrating the stumps. Hemocyte infiltration and proliferation among nerve fibers appears to follow a specific pattern that is correlated with fiber regeneration, suggesting a role for these cells in fostering axonal regrowth. Connective tissue cells also undergo intense proliferation in the nerve, and at 2 weeks post-lesion, these proliferating cells are also positively marked with the neuronal marker NF200, potentially indicating the differentiation of unlabeled stem/progenitor cells (or glial cells). A role for the connective tissue in driving regenerating fibers toward target tissue has also been suggested, resulting in the formation of a spike-like structure in the stump still connected to the brain (Imperadore et al., 2017).

The effect of chromatophore modulation on the skin after denervation was also examined. About 7 days after lesioning, animals at rest are able to produce a homogeneous chromatic pattern on both side of the mantle. Local control exerted by skin receptors was suggested to be involved in the process, as the possibility of target re-innervation can be excluded at such an early stage of regeneration (Imperadore et al., 2017).

### Other tissues and body parts: cornea, lens, brachial gland and brachial hearts

There are only two known accounts of a cephalopod surviving and recovering from lesions to the eyes. A brief appendix is presented in Lange (1920), in which there is mention of the effect of lens extirpation. Survival of animals is greatly affected by surgery, though Lange reports that some animals lived for up to 10 weeks post-surgery (Lange, 1920). Soon after injury, these animals lost the ability to perceive light; the faculty was regained 8 weeks later.

Interestingly, there are two reports of rapid corneal regeneration in two species of octopus, *O. vulgaris* and *Enteroctopus dofleini* (Dingerkus and Santoro, 1981). In the case of *E. dofleini*, the damage had occurred in the wild, with one cornea completely missing. Ten days were required to completely regenerate it, and ultimately, the new cornea was indistinguishable from that of contralateral uninjured eye. To further confirm this finding, the same researchers ablated a single cornea in two *O. vulgaris* females and found that they completely regenerated in 9 and 10 days, respectively. Interestingly, regeneration time was similar for the two species even though they were maintained at very different water temperatures (4–7°C for *E. dofleini* and 22°C for *O. vulgaris*).

At the beginning of the twentieth century, many invertebrate researchers focused on the identification of organs with endocrine functions. Sereni (1932); Mitolo (1938) and Taki (1944) initiated such investigations in cephalopods. They focused on the anatomy and function of the branchial gland in particular, uncovering clues that hinted at an endocrine function (Taki, 1964). These studies reported evidence that the gland often presented signs of necrosis in the animals examined, which apparently was the result of a physiological phenomenon, but that the affected area is continuously replaced by regenerating tissue.

The branchial gland and the branchial heart are also subject to anemic infarct, from which they are able to recover via scar-healing orchestrated by amoebocytes. In the words of the Iwao Taki: “The healing of the infarct is due to the amoebocytes which enter the morbid tissue; they first clean the lesion by devouring the residue tissue, and aggregate together to develop a new tissue. The outer part of the healed tissue is crowded by many fibroblasts containing elongate nuclei, while the inner part is formed by a loose parenchymatous tissue consisting of spherical, undifferentiated cells. In a vigorous animal, the healing proceeds in due course and the secretory activity is resumed” (Taki, 1964, p. 390). In addition, if the function of the branchial gland is suppressed, arm regeneration appears greatly delayed, though never completely inhibited (Taki, 1964).

## Closing remarks

Studies conducted over the last 160 years and summarized here demonstrate the incredible regenerative abilities of cephalopods. Species of cuttlefish, squid and octopus all appear capable of recovering the structure and function of a variety of damaged or lost tissues, including appendages, peripheral nerves, the cornea, and even aspects of the central nervous system. Ultimately, the regenerated tissues are indistinguishable from the original structures.

But, despite the fact that great effort has been expended in the exploration of cephalopod regenerative abilities, the underlying molecular and cellular pathways remain largely uncharacterized. The majority of relevant findings are based on histological analysis, with more recent publications reporting mainly macroscopic and microscopic observations.

Though technical limitations continue to impede attempts to understand regenerative abilities in cephalopods, a number of important findings have been obtained nonetheless.

Among these, one of the most important has been establishment of the role of hemocytes, the circulating cellular components that form the basis of the cephalopod immune system (for review see Gerdol et al., 2018), in various phases of the regeneration process (Lange, 1920; Sereni and Young, 1932; Féral, 1978, 1979, 1988; Polglase et al., 1983; Imperadore et al., 2017). Almost all studies of regeneration in cephalopods report the involvement of hemocytes which rush to the site of the lesion to form a scar, and although this tissue forms a protective plug against pathogens, it does not present a physical barrier to regenerative phenomena (Lange, 1920; Polglase et al., 1983; Féral, 1988). Indeed, in the case of an arm wound, this plug contributes to the formation of the so-called primary blastema, thought to supply material for the regenerating stump (Lange, 1920). A scar also forms between the two stumps of a transected pallial or stellar nerve, but as is the case in non-nervous tissue, a regenerating nerve fiber eventually passes through the scar to re-connect with target tissue.

During healing and regeneration, hemocytes are also involved in removing necrotic tissues by actively phagocytizing debris. They also appear to transdifferentiate into other cell types (Lange, 1920; Féral, 1979, 1988; Polglase et al., 1983). It has been suggested that during arm regeneration, new muscles and nervous cells can only originate from dedifferentiated cells of the same type; by contrast, hemocytes are capable of transforming from round to spindle-shaped (Lange, 1920; Féral, 1979, 1988; Polglase et al., 1983) and apparently to differentiate from fibrocytes (Féral, 1979).

It has also been assumed that chromatophores and iridophores in the skin of a regenerating arm are derived through the dedifferentiation of another cell type, most likely hemocytes or fibrocytes. Both of these cell types have the potential to serve either as chromatophores or iridophores due to their position inside the blastema, close to the epidermis. The possibility that cephalopod hemocytes can transdifferentiate into another cell-type has already been proposed by Jullien et al. (1956), whose findings were later confirmed by Féral's work. However, it must be pointed out that these hypotheses are based only on circumstantial evidence and lack any direct confirmation.

The proliferation of hemocytes during regeneration is another common finding of the studies reviewed here. Early investigations attributed this to amitotic division (Lange, 1920; Sereni and Young, 1932), while more recent accounts noted mitotic cell division (Féral, 1979; Imperadore et al., 2017). This ambiguity might be explained by the different approaches employed: early studies were based only on histology and macroscopic observations with some additional microscopic examination, while more recent work, including that of Imperadore et al. (2017), have benefitted from the use of cellular markers.

Amitosis is a process in which cell division results from nuclear restriction, giving rise to two daughter cells that differ from each other and from the parent cell (e.g., Child, 1907a,b,c,d), because no homogenous segregation of chromosomes occurs (see first description in Remak, 1841 cited in: Lucchetta and Ohlstein, 2017). This process appears to be widespread among invertebrates and vertebrates alike, though its actual function remains unexplained. Recently amitosis has been reported to be involved in stem cell replacement during gut regeneration in *Drosophila melanogaster* (Lucchetta and Ohlstein, 2017).

It is probable that both mitosis and amitosis take place during tissue regeneration in cephalopods as two alternative modes of replenishing degrading tissues and as a source of stem or progenitor cells. However, further investigation is required to elucidate the mechanisms involved.

Lens regeneration and cornea repair have been observed in vertebrates such as newts, frogs and salamanders (e.g., Carinato et al., 2000; Henry and Tsonis, 2010; Henry et al., 2012), but the occurrence of cornea regeneration after complete extirpation has so far only been reported in two species of octopus (*O. vulgaris* and *E. dofleini*, Dingerkus and Santoro, 1981). If documented, widespread occurrence of this ability in octopuses would support their use as models of this phenomenon, leading to further insights that might be applicable even to “higher” vertebrates and human medicine.

Peripheral nerve lesions, which cause severe impairment to affected animals, have also been made in cephalopods in order to observe putative regenerative phenomena. After unilateral pallial nerve transection, animals lose control of breathing muscles and chromatophores on the ipsilateral side of the mantle. Wallerian degeneration is observed in the distal stump of the nerve and chromatolysis is detected in brain cells, as happens also in mammals after nerve injury. However, in the case of cephalopods, nerve regeneration begins a few hours after lesioning, and continues until nerves are reconnected to end target tissues and function is completely recovered. A process of differentiation in stem/progenitor cells thought to enable this regeneration, but this remains speculation (Imperadore et al., 2017).

The potential of this molluscan class to enlighten the study of regeneration is clear, and new tools and techniques that have recently been developed should facilitate its study in the near future.

Despite limited availability of tools allowing more advanced genomic/proteomic analyses, gene function inactivation, and cell labeling, to cite some, researchers are committed in establishing new strategies for the study of regeneration in this taxon.

Label-free multiphoton microscopy (Imperadore et al., 2018) and micro-PET imaging (Zullo et al., 2018) have been recently applied to *O. vulgaris* to follow regeneration after pallial nerve transection (Imperadore et al., 2018) and arm regrowth after amputation (Zullo et al., 2018). The two methods appear very promising: multiphoton microscopy does not rely on any specific marker or dye, allowing the detection of structures and cells usually not revealed with classical staining; micro-PET imaging possibly enable detection of proliferating cells in regenerating tissues and might allow, in the next future, *in vivo* and minimally invasive imaging investigations.

The effort in developing alternative methodologies and/or adapting tools to cephalopod research is very promising and require integration of different scientific communities and fields.

## Author contributions

PI drafted an earlier version of the manuscript. PI and GF contributed to the final writing of the manuscript.

### Conflict of interest statement

The authors declare that the research was conducted in the absence of any commercial or financial relationships that could be construed as a potential conflict of interest.

## Footnotes

^1^http://wokinfo.com/products_tools/specialized/zr/; time-span 1945-2018; last accessed: February, 2018.
